# Diagnostic of FibroTouch and six serological models in assessing the degree of liver fibrosis among patients with chronic hepatic disease: A single-center retrospective study

**DOI:** 10.1371/journal.pone.0270512

**Published:** 2022-07-01

**Authors:** Zhongbao Zuo, Huaizhong Cui, Miaochan Wang, Congxiang Huang, Jing Wu, Chengjing Tao, Zhaoyi Li, Chunli Yang, Kenv Pan, Jianfeng Bao, Shourong Liu, Aifang Xu

**Affiliations:** 1 Department of Clinical Laboratory, Hangzhou Xixi Hospital, Zhejiang, China; 2 Pathology Department, Hangzhou Xixi Hospital, Zhejiang, China; 3 Department of Obstetrics and Gynecology, Hangzhou Xixi Hospital, Zhejiang, China; 4 Science and Education Department, Hangzhou Xixi Hospital, Zhejiang, China; 5 Department of Clinical Research, The 903rd Hospital of PLA, Zhejiang, China; 6 Department of Hepatology, Hangzhou Xixi Hospital, Zhejiang, China; Wakayama Medical University: Wakayama Kenritsu Ika Daigaku, JAPAN

## Abstract

**Background and aims:**

The aim of this study was to evaluate the diagnostic value of FibroTouch and serological models on staging hepatic fibrosis in chronic liver diseases.

**Methods:**

We recruited 850 patients undergoing liver biopsy and received FibroTouch test before or after liver biopsy within one week, blood was taken for the routine inspection before the operation within one week. The serological models were calculated by the blood results and routine clinical information. The diagnostic value of FibroTouch and six serological models was analyzed by receiver operating characteristic curve (ROC).

**Results:**

Patients with severe liver fibrosis had significantly higher AST, ALT, GGT, RDW, ALP, and FT-LSM. The area under the receiver operating characteristic curve (AUROC) of FT-LSM for the liver diagnosis of S≥2, S≥3 and S = 4 was 0.75(95% confidence interval [CI]:0.72–0.78), 0.83(95% CI: 0.80–0.86), and 0.85 (95% CI: 0.81–0.89), respectively. The optimal cut-off of FT-LSM for diagnosing S≥2, S≥3 and S = 4 was 8.7, 10.7, and 12.3, respectively.

**Conclusions:**

Our study showed the FibroTouch has a higher diagnostic value compared with the non-invasive serological models in staging the fibrosis stage. The cut-off of FibroTouch and five serological models (APRI, FIB-4, S-index, Forns, and PRP) increased with the severe of fibrosis stage.

## 1. Introduction

Chronic liver disease (CLD) is affecting millions of people worldwide with increasing mortality and morbidity, and it is still a huge burden for the global health [1]. Liver fibrosis is a pathophysiological process, which refers to the abnormal hyperplasia of connective tissue in liver caused by various pathogenic reasons. Although Chronic hepatitis B virus (CHB) dominates the liver fibrosis [2] in China, the Alcoholic liver disease (ALD), Nonalcoholic fatty liver disease (NAFLD), Drug induced liver injury (DILI), and Autoimmune hepatitis (AIH) are at a rising stage in recent years [2]. CLD leads to progressive liver fibrosis, which could develope into liver cirrhosis and hepatocellular carcinoma. Therefore, early identification of fibrosis grade can effectively prevent to progress disease. However, liver biopsy is still the "gold standard" for the diagnosis of liver fibrosis, which is hardly to complete in the real clinical for its trauma, observer error and sampling error.

FibroTouch, a self-made instrument in China, has achieved good clinical effect which can evaluate the degree of liver fibrosis by detecting liver stiffness measurement (LSM). Previous studies [3, 4] have showed the FibroTouch was an effective tool with high sensitivity and specificity for the diagnosis of liver fibrosis which was comparable with that of Fibroscan. Considering the high cost of Fibroscan, we prefer FibroTouch to stage the degree of fibrosis in our research.

The researchers have tried to explore the serological model of liver fibrosis, such as aspartate transaminase-to-platelet ratio index (APRI), FIB-4, S-Index, Forns, PRP, and Fib-5. APRI is a most common noninvasive diagnostic model for hepatic fibrosis which first applied in HCV patients [5]. FIB-4 first applied in the HCV patients but then extended to other CLD for its simple, widely application, and easy to obtain [6]. Kun Zhou [7] suggested a S-index mathematical model consisting of routine laboratory markers with a high degree of accuracy, possible reducing the need for liver biopsy. Other researchers [8–10] explored some serological models such as Forns, PRR, and Fib-5, which was also effective in the stage of fibrosis. Although the serological models of liver fibrosis can obtain by conventional serological signs, the accurate of the models need to interpret more carefully. The aim of this study was to evaluate the diagnostic value of FibroTouch and serological models on staging hepatic fibrosis in chronic liver diseases.

## 2. Material and methods

### 2.1. Patient

**Fig 1:**
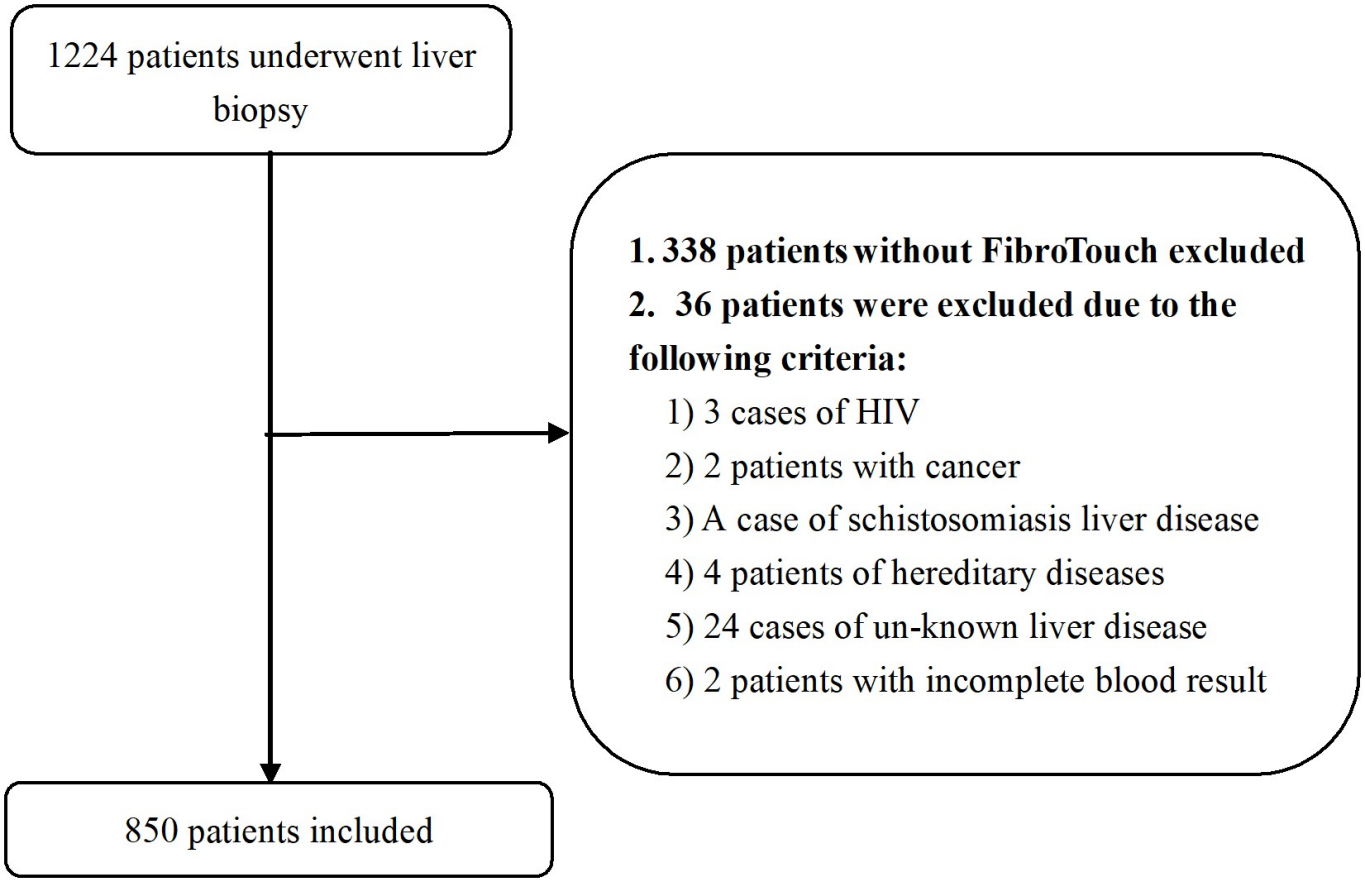
Flow chart of the study design and patient enrollment, 850 patients were included in our research.

Patients who underwent liver biopsy recruited in our analysis from September 1, 2015 to June 10, 2021, and they treated in the hepatology department of Hangzhou Xixi Hospital with CLD. The inclusion criteria were as follows: (1) age older than 16; (2) chronic liver disease; (3) underwent liver biopsy; (4) written informed consent. The exclusion criteria were: (1) co-infection with HIV; (2) cancer; (3) schistosomiasis liver disease; (4) hereditary diseases; (5) un-known liver disease; (6) incomplete information of patients. A flowchart of patient enrollment illustrated in Fig 1. All patients received FibroTouch test before or after liver biopsy within one week, and blood was taken for the routine inspection before the operation within one week. The study approved by the institutional ethics review committee at Hangzhou Xixi Hospital. The study protocol was in accordance with the ethics guidelines of the 1975 Declaration of Helsinki. Written informed consent was unwanted for the retrospective nature of the study (2021 Science Ethic No.02).

### 2.2. Blood test

Alanine aminotransferase (ALT), Aspartate aminotransferase (AST), Glutamyl transpeptidase (GGT), Cholesterol (CHO), Albumin (ALB), and Alkaline phosphatase (ALP) tested by the Beckman Coulter au5831 automatic biochemical analyzer at laboratory department in Xixi hospital. Platelet (PLT) and Red Cell volume Distribution Width (RDW) detected using the automatic blood cell analyzer.

### 2.3. FibroTouch

During the measurement, the patient was in the supine position, right hand raised and placed on the head, and the detection points were selected from the right axillary front to the 7th, 8th and 9th ribs of the axillary midline, close to the rib space. The FibroTouch liver stiffness (FT-LSM) value was measured at a fitting time for 10 consecutive effective tests. Finally, the median was taken as the F value to mark the degree of fibrosis of liver tissue, The results with deviation greater than one third of the median data and operation success rate less than 60% considered invalid. All operations were completed by the same doctor who had received professional training.

### 2.4. Histopathological staging of liver fibrosis

After being reviewed and approved by the ethics committee of Hangzhou Xixi Hospital, the patients signed the informed consent form and used the ultrasound-guided adjustable ejection biopsy gun for percutaneous liver biopsy. The length of liver tissue was 1.5–2.0 cm, fixed with 4% formaldehyde, embedded in paraffin, and sectioned with a thickness of 5 um for HE and Masson staining. The liver tissue section contained at least 6 portal areas. Hepatic fibrosis performed by two pathologists in a double-blind manner using the Scheuer scoring system [3, 11], S0: no fibrosis; S1: fibrosis enlarged in the portal tracts; S2: fibrous septum formation in the portal area, but intact architecture; S3: fibrosis with lobular structure disorder, but no obvious cirrhosis; S4: cirrhosis.

### 2.5. The calculation formula of serological model


APRI=AST/ULN*100/PLT;

$$\text{FIB-}4=\text{age}*\text{AST}/\left(\text{PLT}*\surd \text{ALT}\right);$$

S-Index=1000*GGT/(PLT*ALB^2);

$$\text{Forns}=7.811-3.131*\text{ln}\left(\text{PLT}\right)+0.781*\text{ln}\left(\text{GGT}\right)+3.467*\text{ln}\left(\text{age}\right)-0.014*\text{CHO};$$

PRP=RDW/PLT;

$$\text{Fib-}5=0.3*\text{ALB}+0.05*\text{PLT}-0.014*\text{ALP}+6*\left(\text{AST}/\text{ALT}\right)+14.$$

### 2.6. statistical analysis

SAS 9.4 (SAS Institute Inc., Cary, NC) statistical software was used for analysis. The continuous skewed data were expressed by median (interquartile range, IQR). Spearman correlation analysis was used for the correlation between fibrosis stage and other evaluation methods. The diagnostic value of FibroTouch and six serological models was analyzed by receiver operating characteristic curve (ROC). The closer of AUROC to 1, the better diagnostic value it can provide. The comparison of AUROC among different models and FT-LSM was analyzed by the Delong test. P value < 0.05 considered as statistically significant for all the tests.

## 3. Results

### 3.1. Patient characteristics

A total of 850 CLD patients who underwent liver biopsy, received FibroTouch measurement and blood test included in the final analysis, and the detail information were shown in Table 1. There were 344 patients with mild fibrosis or no fibrosis (S<2), including 206 males and 138 females, with a median age of 42.5 years.

**Table 1:** The characteristics of 850 chronic liver disease patients from 2016 to 2021, Hangzhou, Zhejiang, China.

Characteristics	All (850)	S<2(344)	2≤S<4(410)	S = 4(96)	P
Age, years	44.0(34.0–52.0)	42.5(33.5–50.0)	45.0(35.0–52.0)	46.0(38.0–56.0)	0.002
Gender					0.0003
Male, n (%)	471 (55.4)	206(59.5)	200(48.8)	65(67.7)	
Female, n (%)	379 (44.6)	138(40.1)	210(51.2)	31(32.3)	
Chronic liver disease					0.11
CHB, n (%)	677 (79.6)	265(77.0)	329(80.2)	83(86.5)	
others, n (%)	173 (20.4)	79(23.0)	81(19.8)	13(13.5)	
Serum test					
AST (U/L), median (IQR)	34(26–48)	29(23–38)	37(27–56)	41(31–56)	<0.0001
ALT (U/L), median (IQR)	42(27–67)	38(24–58)	46(29–73)	42(30–57)	0.0002
GGT (U/L), median (IQR)	33(21–62)	27(18–52)	36(22–66)	50(27–77)	<0.0001
ALB (g/L), median (IQR)	41.8(38.0–44.9)	42.9(39.7–45.6)	41.5(37.7–44.5)	38.1(33.6–43.5)	<0.0001
CHO (mmol/L), median (IQR)	4.5(3.9–5.2)	4.6(4.0–5.2)	4.5(3.9–5.2)	4.1(3.4–4.8)	<0.0001
RDW (100%), median (IQR)	0.13(0.12–0.13)	0.13(0.12–0.13)	0.13(0.12–0.14)	0.13(0.13–0.14)	<0.0001
PLT (10^9/L), median (IQR)	185.0(148.0–224.0)	204.0(168.0–136.5)	179.5(144.0–218.0)	133.5(94.0–171.5)	<0.0001
ALP (U/L), median (IQR)	95.0(77.0–118.0)	93.0(75.0–114.0)	94.0(78.0–117.0)	104.5(86.5–130.5)	<0.0001
FT-LSM, median (IQR)	8.9(6.4–12.5)	7.1(5.7–9.1)	9.6(7.4–12.9)	15.3(12.5–20.9)	<0.0001
Serological model					
APRI, median (IQR)	0.54(0.38–0.86)	0.42(0.31–0.59)	0.61(0.44–0.93)	0.84(0.59–1.75)	<0.0001
FIB-4, median (IQR)	1.28(0.83–1.99)	1.04(0.73–1.42)	1.41(0.92–2.13)	2.22(1.48–4.21)	<0.0001
S-index, median (IQR)	0.11(0.06–023)	0.07(0.05–0.14)	0.12(0.07–0.23)	0.24(0.13–0.58)	<0.0001
Forns, median (IQR)	7.24(6.00–8.53)	6.74(5.64–7.77)	7.44(6.09–8.62)	8.69(7.45–9.80)	<0.0001
FIB-5, median (IQR)	39.79(37.19–42.52)	40.68(38.23–43.55)	39.27(36.95–42.14)	37.27(34.28–39.47)	<0.0001
PRP, median (IQR)	0.07(0.06–0.09)	0.06(0.05–0.08)	0.07(0.06–0.09)	0.10(0.08–0.14)	<0.0001

Abbreviations: Aspartate aminotransferase: AST; Alanine aminotransferase: ALT; Glutamyl transpeptidase: GGT; Albumin: ALB; Cholesterol: CHO; Red Cell volume Distribution Width: RDW; Platelet: PLT; Alkaline phosphatase: ALP; FibroTouch liver stiffness: FT-LSM; Aspartate transaminase-to-platelet ratio index: APRI; Interquartile Range: IQR.

410 patients had moderate to severe fibrosis (2≤S<4), including 200 males and 210 females, with a median age of 45.0 years. 96 patients had cirrhosis, with a median age of 46.0 years. There were no significant differences between chronic HBV disease and other CLD, but age and gender were significantly different among different grades of liver fibrosis. Patients with severe liver fibrosis had significantly higher AST, ALT, GGT, RDW, ALP, and FT-LSM; a significantly lower ALB, CHO, and PLT was seen in the high grade of liver fibrosis. All the serological models were significantly different among different grades of hepatic fibrosis, and only the FIB-5 negative correlated with the liver fibrosis (Table 1).

### 3.2. Correlations among fibrosis stage, serological model, and FT-LSM

We used the fibrosis stage, serological models, and FT-LSM to build a Spearman’s correlation coefficient in Table 2. All the correlations between any two were statistically significant, but the Forns and FIB-4 strongly correlated (r = 0.83, P<0.0001). The fibrosis stage had the biggest correlation with FT-LSM (r = 0.54, P<0.0001). FIB-5 negative correlated with other serological models, but the remain correlation coefficient was positive.

**Table 2:** The correlation among fibrosis stage, serological model, and FT-LSM.

Variable	FT-LSM	Fibrosis stage	APRI	FIB-4	S-Index	Forns	PRP	FIB-5
FT-LSM	1	0.54*	0.35*	0.33*	0.44*	0.34*	0.28*	-0.25*
Fibrosis stage	0.54*	1	0.42*	0.39*	0.36*	0.32*	0.35*	-0.26*
APRI	0.35*	0.42*	1	0.68*	0.57*	0.54*	0.60*	-0.54*
FIB-4	0.33*	0.39*	0.68*	1	0.47*	0.83*	0.69*	-0.39*
S-Index	0.44*	0.36*	0.57*	0.47*	1	0.71*	0.46*	-0.57*
Forns	0.34*	0.32*	0.54*	0.83*	0.71*	1	0.71*	-0.60*
PRP	0.28*	0.35*	0.60*	0.69*	0.46*	0.71*	1	-0.73*
FIB-5	-0.25*	-0.26*	-0.54*	-0.39*	-0.57*	-0.60*	-0.73*	1

*: Statistically significant, P<0.0001.

Abbreviations: FibroTouch liver stiffness: FT-LSM; Aspartate transaminase-to-platelet ratio index: APRI.

### 3.3. Performance of serological model and FT-LSM in fibrosis stage assessment

**Fig 2:**
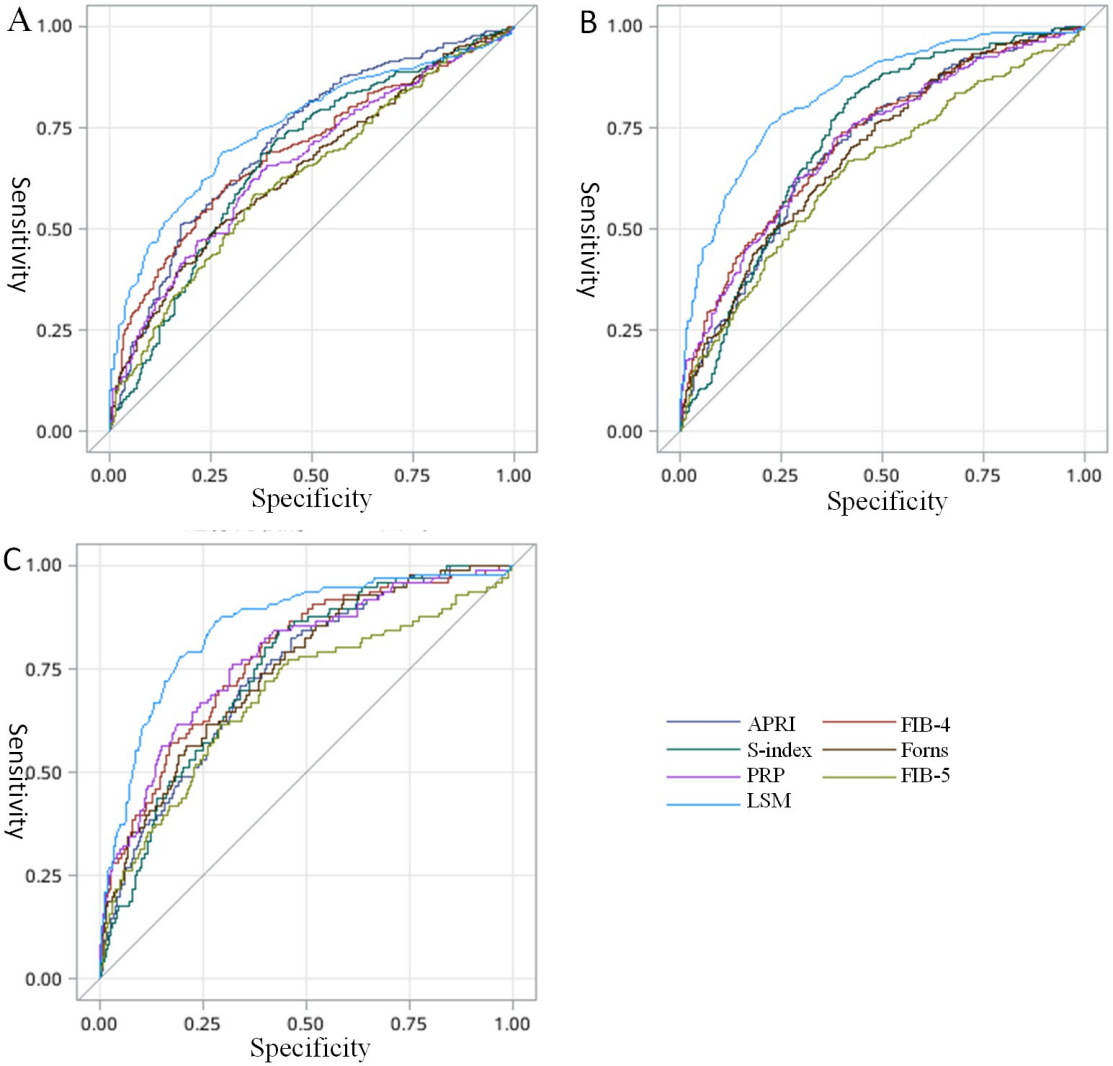
Effects of FibroTouch and six serological models on the staging of liver fibrosis using ROC curve. A: ROC curve of patients with liver histopathological stage S ≥ 2; B: ROC curve of patients with liver histopathological stage S ≥ 3; C: ROC curve of patients with liver histopathological stage S = 4.

The performance of serological model and FT-LSM in assessing fibrosis stage was shown in Table 3 and Fig 2. The area under the receiver operating characteristic curve (AUROC) of FT-LSM under the diagnosis of S≥2, S≥3 and S = 4 was 0.75(95% confidence interval [CI]:0.72–0.78), 0.83(95% CI: 0.80–0.86), and 0.85 (95% CI: 0.81–0.89), respectively. The optimal cut-off of FT-LSM for diagnosing S≥2, S≥3 and S = 4 was 8.7, 10.7, and 12.3, respectively. For fibrosis S≥2, the AUROC was 0.72 for APRI (APRI vs LSM, P = 0.15), 0.69 for FIB-4 (FIB-4 vs LSM, P = 0.008), 0.67 for S-index (S-index vs LSM, P = 0.0002), 0.64 for Forns (Forns vs LSM, P<0.0001), 0.66 for PRP (PRP vs LSM, P = 0.0001), and 0.63 for FIB-5 (FIB-5 vs LSM, P<0.0001), respectively. For fibrosis S≥3, the AUROC was 0.70 for APRI (APRI vs LSM, P P<0.0001), 0.72 for FIB-4 (FIB-4 vs LSM, P<0.0001), 0.73 for S-index (S-index vs LSM, P<0.0001), 0.69 for Forns (Forns vs LSM, P<0.0001), 0.71 for PRP (PRP vs LSM, P<0.0001), and 0.64 for FIB-5 (FIB-5 vs LSM, P<0.0001), respectively. For fibrosis S = 4, the AUROC was 0.74 for APRI (APRI vs LSM, P = 0.0003), 0.78 for FIB-4 (FIB-4 vs LSM, P = 0.02), 0.75 for S-index (S-index vs LSM, P = 0.0001), 0.75 for Forns (Forns vs LSM, P = 0.001), 0.78 for PRP (PRP vs LSM, P = 0.03), and 0.68 for FIB-5 (FIB-5 vs LSM, P<0.0001), respectively. We did see a rising AUROC trend among FT-LSM, FIB-4, S-index, Forns, PRP, and FIB-5, but the APRI was rising in fibrosis S≥3 and then falling in S = 4 which was different with the other model.

**Table 3:** The diagnostic value of FibroTouch and six serological models to fibrosis S≥2, S≥3 and S = 4.

Variable	AUC	95% CI	Cut-off	Specificity	Sensitivity
S≥2					
FT-LSM	0.75	0.72–0.78	8.7	0.718	0.692
APRI^#^	0.72	0.68–0.75	0.64	0.817	0.546
FIB-4*	0.69	0.66–0.73	0.31	0.701	0.619
S-index*	0.67	0.63–0.71	0.095	0.628	0.684
Forns*	0.64	0.61–0.68	7.68	0.738	0.502
PRP*	0.66	0.62–0.70	0.07	0.645	0.625
FIB-5*	0.63	0.59–0.66	39.68	0.645	0.583
S≥3					
FT-LSM	0.83	0.80–0.86	10.7	0.788	0.742
APRI*	0.70	0.66–0.74	0.64	0.705	0.627
FIB-4*	0.72	0.68–0.76	1.33	0.610	0.728
S-index*	0.73	0.69–0.76	0.10	0.594	0.811
Forns*	0.69	0.65–0.73	7.3	0.583	0.696
PRP*	0.71	0.67–0.75	0.073	0.618	0.724
FIB-5*	0.64	0.60–0.69	39.52	0.586	0.668
S = 4					
FT-LSM	0.85	0.81–0.89	12.3	0.806	0.781
APRI*	0.74	0.69–0.79	0.63	0.642	0.729
FIB-4*	0.78	0.73–0.83	1.4	0.609	0.812
S-index*	0.75	0.70–0.80	0.11	0.565	0.844
Forns*	0.75	0.70–0.80	8.2	0.741	0.615
PRP*	0.78	0.73–0.83	0.08	0.678	0.760
FIB-5*	0.68	0.62–0.75	37.95	0.710	0.615

^#^: comparing with FT-LSM, no statistical significance;

*: comparing with FT-LSM, statistically significant.

Abbreviations: FibroTouch liver stiffness: FT-LSM; Aspartate transaminase-to-platelet ratio index: APRI.

## 4. Discussion

**Fig 3:**
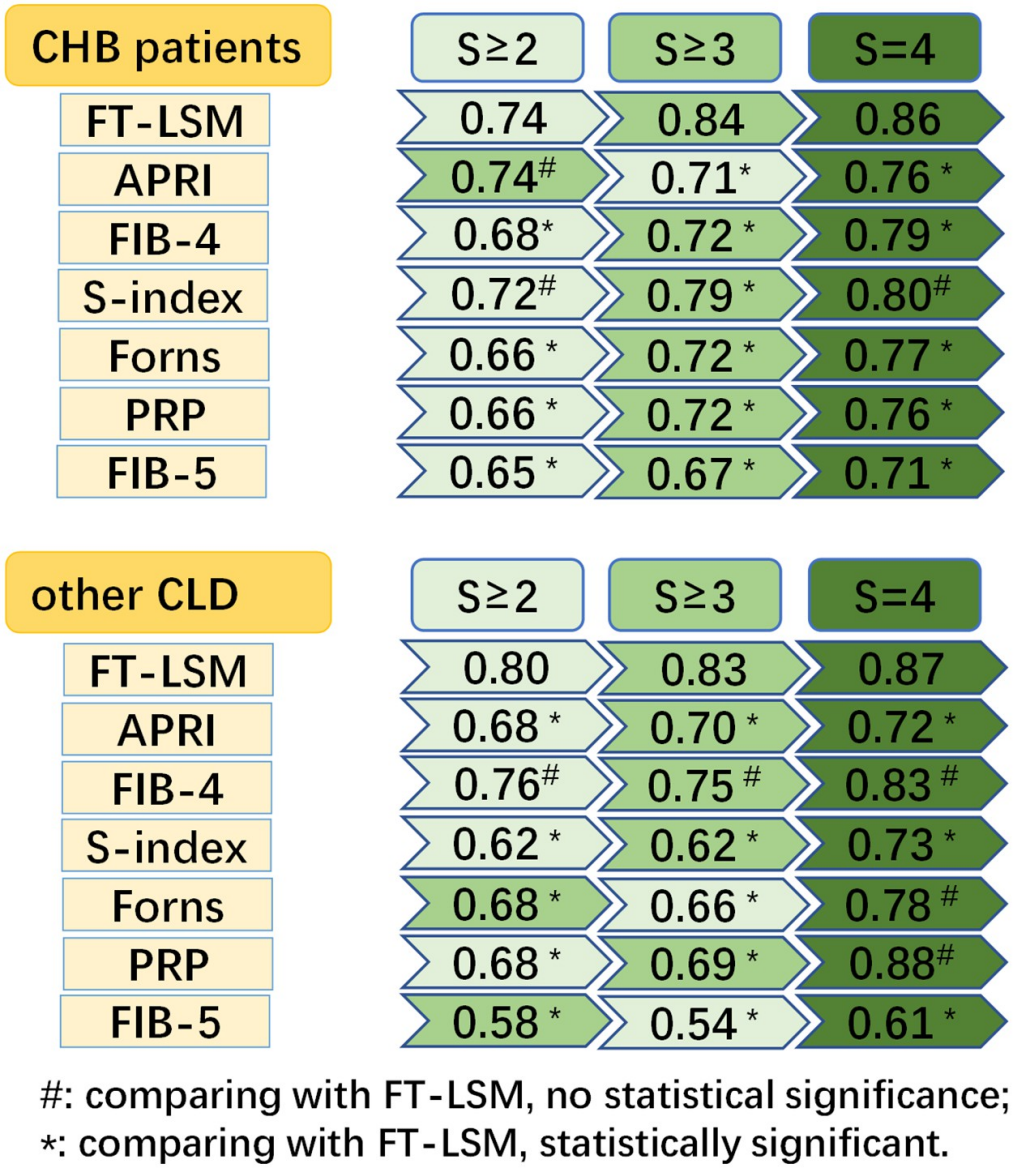
AUROC between CHB patients and other CLD patients among different fibrosis stage to FibroTouch and six serological models. FT-LSM: FibroTouch liver stiffness; APRI: Aspartate transaminase-to-platelet ratio index.

A total of 850 patients with chronic liver disease enrolled in this single-center retrospective study. The FibroTouch provided better performance with AUROC values of 0.75, 0.83, and 0.85 in diagnosing significant fibrosis, severe fibrosis, and cirrhosis, respectively, which was significantly higher than that with other serological models (except S≥ 2 group, comparing with APRI, no statistical significance). The good performance in staging the hepatic fibrosis also found in other researchers [3, 4, 12] with small sample sizes (39–432), but our study enrolled 850 CLD patients to explore the accuracy of the FibroTouch, which was more reliable to evaluate of the diagnostic value. We also found the AUROC of FibroTouch increased with the severe of liver fibrosis in both CHB patients and the other CLD patients (Fig 3). The cut-off of FibroTouch and five serological models (APRI, FIB-4, S-index, Forns, and PRP) increased with the severe of fibrosis stage, but the FIB-5 decreased with that (Table 3), which was a good sign for grading the fibrosis.

The early diagnosis and accurate evaluation of liver fibrosis is helpful to select clinical treatment, evaluation and prognosis of patients with chronic hepatitis. Liver biopsy is still the gold standard for fibrosis grading, but patients undergoing biopsy often feel discomfort and risk of complications, with a pooled rate of adverse events of 2.3% [13]. At the same time, some tissues biopsied cannot represent the fibrosis of the whole liver. The researchers mainly focused on the noninvasive diagnosis of liver fibrosis in recent years, and they had set up some conventional serological index models. However, these models had different abilities in judging liver fibrosis. Some studies believed that the diagnostic value of FIB-4 [14] (AUC = 0.81, P < 0.001) was better than APRI, while others [6] believed that FIB-4 and APRI were consistent in judging liver fibrosis. This study found the AUROCs of APRI in S ≥ 2 (0.72), S-index in S ≥ 3 (0.73), and FIB-4/PRP in S = 4 (0.77) were higher than the other serological models, which is consistent with the results the serological models may have different diagnostic effects to the stage of liver fibrosis [15]. Interestingly, the AUROCs of APRI were 0.72 in fibrosis S≥2, 0.70 in S≥3, 0.74 in S = 4, respectively, with a ‘first down and then up’ trend in the general CLD patients (Table 3), which also appeared in the CHB patients (Fig 3). However, the serological model FIB-4, Forns, and FIB-5 were having a ‘first down and then up’ trend (Fig 3) in the other CLD patients. The possible explain for the different serological model trend among CLD patients may attribute to the different progression of disease, such as autoimmune hepatitis [16], Nonalcoholic Fatty Liver Disease [17], and drug-induced liver disease [18].

The hepatic fibrosis of CLD was often accompanied by liver dysfunction and impaired synthesis of coagulation factors. Our study showed the Patients with severe liver fibrosis had significantly higher AST, ALT, GGT, and ALP, and a significantly lower ALB, CHO, and PLT was also seen in the high grade of liver fibrosis. Previous researchers [9, 19] also found the severe liver fibrosis has worse liver dysfunction. Our study found the FIB-5 negative correlated with fibrosis stage, which also found in other researches [10, 20]. Although the positive correlation (except FIB-5 group) among different serological model and fibrosis stage was not that large, we did see a significantly difference among different grade of fibrosis stage (Table 1).

## 5. Conclusion

Our study showed the FibroTouch has a higher diagnostic value compared with the non-invasive serological models in staging the fibrosis stage. The cut-off of FibroTouch and five serological models (APRI, FIB-4, S-index, Forns, and PRP) increased with the severe of fibrosis stage, but the FIB-5 decreased with the cut-off. We also found the serological models have different diagnostic effects to the stage of liver fibrosis between CHB patients and other CLD patients.

## References

[1] Collaborators GCoD (2018) Global, regional, and national age-sex-specific mortality for 282 causes of death in 195 countries and territories, 1980–2017: a systematic analysis for the Global Burden of Disease Study 2017. Lancet 392: 1736–1788. doi: 10.1016/S0140-6736(18)32203-7 30496103 PMC6227606

[2] Xiao J, Wang F, Wong NK, He J, Zhang R, et al. (2019) Global liver disease burdens and research trends: Analysis from a Chinese perspective. J Hepatol 71: 212–221. doi: 10.1016/j.jhep.2019.03.004 30871980

[3] Xu Y, Liu Y, Cao Z, Wang L, Li Z, et al. (2019) Comparison of FibroTouch and FibroScan for staging fibrosis in chronic liver disease: Single-center prospective study. Dig Liver Dis 51: 1323–1329. doi: 10.1016/j.dld.2019.02.009 30928419

[4] Serra JT, Mueller J, Teng H, Elshaarawy O, Mueller S (2020) Prospective Comparison of Transient Elastography Using Two Different Devices: Performance of FibroScan and FibroTouch. Hepat Med 12: 41–48. doi: 10.2147/HMER.S245455 32280285 PMC7125402

[5] Wai CT, Greenson JK, Fontana RJ, Kalbfleisch JD, Marrero JA, et al. (2003) A simple noninvasive index can predict both significant fibrosis and cirrhosis in patients with chronic hepatitis C. Hepatology 38: 518–526. doi: 10.1053/jhep.2003.50346 12883497

[6] Wang H, Xue L, Yan R, Zhou Y, Wang MS, et al. (2013) Comparison of FIB-4 and APRI in Chinese HBV-infected patients with persistently normal ALT and mildly elevated ALT. J Viral Hepat 20: e3–10. doi: 10.1111/jvh.12010 23490387

[7] Zhou K, Gao CF, Zhao YP, Liu HL, Zheng RD, et al. (2010) Simpler score of routine laboratory tests predicts liver fibrosis in patients with chronic hepatitis B. J Gastroenterol Hepatol 25: 1569–1577. doi: 10.1111/j.1440-1746.2010.06383.x 20796157

[8] Smas M, Skubera M, Wilkosz T, Werynski P, Kolcz J, et al. (2019) Noninvasive assessment of liver status in adult Fontan patients. Pol Arch Intern Med. 10.20452/pamw.4452 30778020

[9] Yuyun D, Zhihua T, Haijun W, Zhaoping L, Xiaoli Z, et al. (2019) Predictive value of the red blood cell distribution width-to-platelet ratio for hepatic fibrosis. Scand J Gastroenterol 54: 81–86. doi: 10.1080/00365521.2018.1558786 30663454

[10] Shiha G, Seif S, Eldesoky A, Elbasiony M, Soliman R, et al. (2017) A simple bedside blood test (Fibrofast; FIB-5) is superior to FIB-4 index for the differentiation between non-significant and significant fibrosis in patients with chronic hepatitis C. Hepatol Int 11: 286–291. doi: 10.1007/s12072-017-9796-z 28425016

[11] Yang XZ, Gen AW, Xian JC, Xiao L (2018) Diagnostic value of various noninvasive indexes in the diagnosis of chronic hepatic fibrosis. Eur Rev Med Pharmacol Sci 22: 479–485. doi: 10.26355/eurrev_201801_14198 29424906

[12] Qu Y, Song YY, Chen CW, Fu QC, Shi JP, et al. (2021) Diagnostic Performance of FibroTouch Ultrasound Attenuation Parameter and Liver Stiffness Measurement in Assessing Hepatic Steatosis and Fibrosis in Patients With Nonalcoholic Fatty Liver Disease. Clin Transl Gastroenterol 12: e00323. doi: 10.14309/ctg.0000000000000323 33848277 PMC8049161

[13] Mohan BP, Shakhatreh M, Garg R, Ponnada S, Adler DG (2019) Efficacy and safety of EUS-guided liver biopsy: a systematic review and meta-analysis. Gastrointest Endosc 89: 238–246.e233. doi: 10.1016/j.gie.2018.10.018 30389469

[14] Liu XD, Wu JL, Liang J, Zhang T, Sheng QS (2012) Globulin-platelet model predicts minimal fibrosis and cirrhosis in chronic hepatitis B virus infected patients. World J Gastroenterol 18: 2784–2792. doi: 10.3748/wjg.v18.i22.2784 22719186 PMC3374981

[15] Cheng J, Hou J, Ding H, Chen G, Xie Q, et al. (2015) Validation of Ten Noninvasive Diagnostic Models for Prediction of Liver Fibrosis in Patients with Chronic Hepatitis B. PLoS One 10: e0144425. doi: 10.1371/journal.pone.0144425 26709706 PMC4692502

[16] Xing X, Yan Y, Shen Y, Xue M, Wang X, et al. (2020) Liver fibrosis with two-dimensional shear-wave elastography in patients with autoimmune hepatitis. Expert Rev Gastroenterol Hepatol 14: 631–638. doi: 10.1080/17474124.2020.1779589 32510248

[17] Castera L, Friedrich-Rust M, Loomba R (2019) Noninvasive Assessment of Liver Disease in Patients With Nonalcoholic Fatty Liver Disease. Gastroenterology 156: 1264–1281.e1264. doi: 10.1053/j.gastro.2018.12.036 30660725 PMC7505052

[18] Ma X, Zhou Y, Qiao B, Jiang S, Shen Q, et al. (2020) Androgen aggravates liver fibrosis by activation of NLRP3 inflammasome in CCl(4)-induced liver injury mouse model. Am J Physiol Endocrinol Metab 318: E817–e829. doi: 10.1152/ajpendo.00427.2019 32182125

[19] Wang J, Pu Y, Gong Y, Li Z, Zhu X (2020) A statistical analysis of the correlations among various types of clinical indexes for patients with chronic hepatitis B: A hospital-based study. Medicine (Baltimore) 99: e19201. 32080106 10.1097/MD.0000000000019201 PMC7034703

[20] Kolhe KM, Amarapurkar A, Parikh P, Chaubal A, Chauhan S, et al. (2019) Aspartate transaminase to platelet ratio index (APRI) but not FIB-5 or FIB-4 is accurate in ruling out significant fibrosis in patients with non-alcoholic fatty liver disease (NAFLD) in an urban slum-dwelling population. BMJ Open Gastroenterol 6: e000288. doi: 10.1136/bmjgast-2019-000288 31275584 PMC6577364

